# Deletion Mutants of VPg Reveal New Cytopathology Determinants in a Picornavirus

**DOI:** 10.1371/journal.pone.0010735

**Published:** 2010-05-20

**Authors:** Armando Arias, Celia Perales, Cristina Escarmís, Esteban Domingo

**Affiliations:** 1 Departamento de Virología y Microbiología, Centro de Biología Molecular “Severo Ochoa” (CSIC-UAM), Consejo Superior de Investigaciones Científicas (CSIC), Campus de Cantoblanco, Madrid, Spain; 2 Centro de Investigación Biomédica en Red de Enfermedades Hepáticas y Digestivas (CIBERehd), Barcelona, Spain; The University of Chicago, United States of America

## Abstract

**Background:**

Success of a viral infection requires that each infected cell delivers a sufficient number of infectious particles to allow new rounds of infection. In picornaviruses, viral replication is initiated by the viral polymerase and a viral-coded protein, termed VPg, that primes RNA synthesis. Foot-and-mouth disease virus (FMDV) is exceptional among picornaviruses in that its genome encodes 3 copies of VPg. Why FMDV encodes three VPgs is unknown.

**Methodology and Principal Findings:**

We have constructed four mutant FMDVs that encode only one VPg: either VPg_1_, VPg_3_, or two chimeric versions containing part of VPg_1_ and VPg_3_. All mutants, except that encoding only VPg_1_, were replication-competent. Unexpectedly, despite being replication-competent, the mutants did not form plaques on BHK-21 cell monolayers. The one-VPg mutant FMDVs released lower amounts of encapsidated viral RNA to the extracellular environment than wild type FMDV, suggesting that deficient plaque formation was associated with insufficient release of infectious progeny. Mutant FMDVs subjected to serial passages in BHK-21 cells regained plaque-forming capacity without modification of the number of copies of VPg. Substitutions in non-structural proteins 2C, 3A and VPg were associated with restoration of plaque formation. Specifically, replacement R55W in 2C was repeatedly found in several mutant viruses that had regained competence in plaque development. The effect of R55W in 2C was to mediate an increase in the extracellular viral RNA release without a detectable increase of total viral RNA that correlated with an enhanced capacity to alter and detach BHK-21 cells from the monolayer, the first stage of cell killing.

**Conclusions:**

The results link the VPg copies in the FMDV genome with the cytopathology capacity of the virus, and have unveiled yet another function of 2C: modulation of picornavirus cell-to-cell transmission. Implications for picornaviruses pathogenesis are discussed.

## Introduction

**Figure 1:**
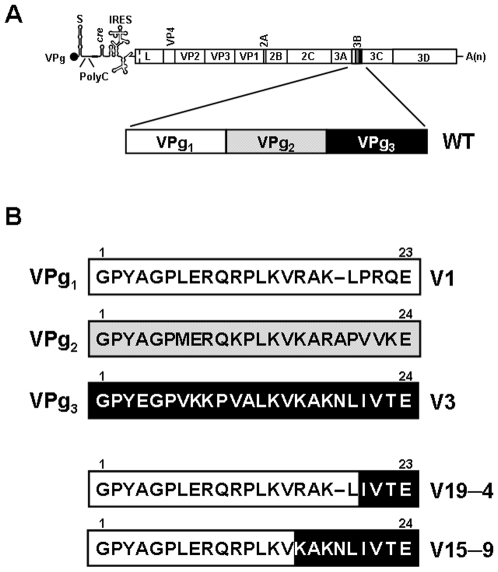
Schematic representation of the FMDV genome and of the constructions with one copy of VPg. **A**, FMDV genome (VPg is the protein covalently linked to the 5′–end of the RNA; PolyC is the internal polycytidylate tract; IRES is the internal ribosome entry site; A(n) is the PolyA at the 3′–end). The genomic region encoding the viral polyprotein is boxed. The viral polyprotein is processed into the different mature proteins indicated in each corresponding box (based in [15]). Gene 3B (highlighted) encodes 3 different but related copies of FMDV protein–primer VPg. **B**, Amino acid sequence of VPg_1_, VPg_2_ and VPg_3_ of FMDV C–S8c1. Infectious FMDV clones were constructed either to express VPg_1_ (V1), VPg_3_ (V3), a chimeric VPg consisting of the first 19 residues from VPg_1_ (N-terminus) and the last 4 residues from VPg_3_ (C-terminus) (V19–4), or a chimeric VPg containing the first 15 residues from VPg_1_ and the last 9 residues from VPg_3_ (V15–9). The starting pMT28 plasmid and procedures for the construction are detailed in Materials and Methods.

Contrary to initiation of cellular DNA replication which is primed by RNA molecules synthesised by cellular primases [1], viruses use a wide variety of molecular mechanisms to initiate genome replication, that include *de novo* initiation, priming by proteins or by self generated 3′–ends of templates, and ‘cap–snatching’, among other mechanisms [2]. Protein–primed initiation of genome replication is used by several DNA and RNA viruses and some linear plasmids [3]–[5]. *Picornaviridae* is a family of positive strand RNA viruses that use as protein–primer a small peptide of about 20 residues in length, termed VPg or 3B [3], [6], [7]. After replication, the protein–primer VPg remains bound to the genomic RNA encapsidated into viral particles. Picornaviruses encode only one copy of VPg, except foot–and–mouth disease virus (FMDV) that expresses three similar but non–identical copies of VPg (VPg_1–3_ or 3B_1–3_) [8] (Figure 1). Each of the three VPgs are found covalently bound to genomic viral RNA [9] and they can be uridylylated *in vitro* by the viral polymerase, with VPg_3_>VPg_2_>VPg_1_ as the order of substrate efficiency [10]. The biological meaning of this unique in–tandem repetition in an RNA virus is not well understood [11], [12]. Molecular poliovirus clones constructed to express two VPgs delete one of the two copies, and the polyprotein harboring two VPgs underwent aberrant processing [13], [14]. FMDV encoding only VPg_3_ is infectious in cell culture, showing that one copy of VPg may be sufficient to complete the virus replication cycle [12]. The virus expressing only VPg_3_ was infectious for hamster and bovine fibroblasts (BHK and FBK cells), but not swine fibroblasts (FPK cells), and was attenuated for swine [12]. FMDVs encoding VPg_1_ and VPg_2_, but lacking VPg_3_ were not viable, suggesting that the presence of VPg_3_ was essential for FMDV viability [11]. The authors proposed that this loss of viability could be due to a defect in the proteolytic processing of the viral polyprotein precursor lacking VPg_3_ [11].

Picornaviral proteins are generated by proteolytic processing of a single viral polyprotein which is translated from a single ORF. During and after translation, different cleavages of the viral polyprotein take place, most of them catalysed by the viral protease 3C, resulting in the release of different processing intermediates and mature proteins (reviewed in [15]). Specifically, the capsid precursor (P1) is processed into VP0 (VP4–VP2), VP3 and VP1 which are assembled to form the mature virions. P2 and P3 precursors render non-structural proteins 2A, 2B, 2C, 3A, 3B (VPg), 3C, 3D and several processing intermediates which are required for viral replication. 3D is the viral RNA-dependant RNA polymerase (RdRp) that catalyses genomic RNA synthesis and the critical VPg–uridylylation step at the initiation of replication. 3C and its precursor 3CD stimulate the initial VPg–uridylylation step, an activity additional to their role in polyprotein processing [16]–[18]. It has been recently proposed that a precursor form of VPg (either 3AB or 3BC) could act as the authentic protein–primer molecule, while processing and release of 3B (VPg) would be a step subsequent to initiation of replication, although these hypotheses are still under discussion [16], [17], [19]. 2C and 3A play also central roles in picornavirus replication. 2C includes NTPase and RNA–binding activities [20]–[23], acts as an RNA chaperone during picornaviral replication [24], and is involved in viral RNA encapsidation [25], uncoating [26], and in host cell membrane rearrangements required for replication [27]–[31]. 3A is a membrane protein [32] that can establish interactions with 2C [33], suggesting that 3A and 2C may constitute part of the same protein complexes for some biological processes. In addition, 3AB precursor (3A bound to VPg) may be involved in the recruitment of 3D polymerase to membranes to form replication complexes in which the viral genomes are synthesised [34], [35].

In the present report, we provide evidence of a functional link between VPg and 2C in the release of FMDV from cells. We have constructed FMDVs that encode a single VPg. Some mutants were replication–competent but did not produce plaques on BHK–21 cell monolayers. Passage of these viruses in BHK–21 cells selected for mutants in 2C, 3A and 3B (VPg) that regained the ability to develop plaques. These mutants displayed increased cytopathology and virus shedding into the extracellular medium. The results provide evidence for a function of the triplicated VPg in the detachment of cells from the monolayers, which is the event that precedes cell killing by FMDV [36], [37]. These observations establish functional connections between VPg and non–structural FMDV proteins.

## Results

### FMDVs with only one VPg copy are replication–competent viruses

**Figure 2:**
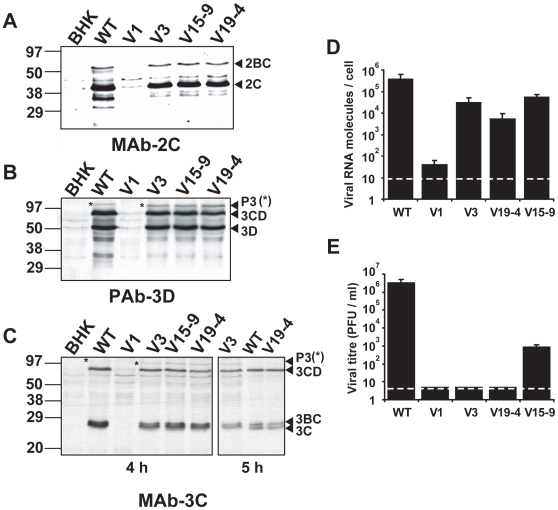
Replication of mutant FMDVs in BHK–21 cells. **A–C**, Western blot assays for the specific detection of FMDV proteins using monoclonal antibody (MAb) 1C8 specific for 2C (panel A), a polyclonal antibody (PAb) specific for 3D (panel B), and MAb 2D2 specific for 3C (panel C). A total of 10^6^ BHK–21 cells were mock–electroporated (BHK lane) or electroporated with either 2 µg of WT RNA or 8 µg of the mutant transcript indicated in each lane. A 4–fold excess of transcript from mutant plasmids was required to reach a comparable level of viral proteins for WT and mutant RNAs. At 4 hours post–electroporation cells were collected in lysis buffer, subjected to SDS–PAGE, and then transferred to a nitrocellulose membrane. The MAbs and the PAb have been previously described [62]. Molecular size markers were run in parallel and their position is indicated on the left. The positions of viral proteins 2BC, 2C, P3, 3CD, 3BC and 3C, determined with specific MAbs, are indicated [62]. Note that precursor P3 (3ABCD, highlighted with an asterisk) displayed a higher mobility than WT in all the mutant transcripts, consistent with a decrease of the number of VPg copies. The right panel in C, (lanes V3, WT and V19–4) correspond to the analysis of cells collected at 5 h instead of 4 h post–transfection, and is included here because of easier band identification. **D**, Average number of viral RNA molecules per cell (quantitated both inside the cells and in the culture medium) at 72 hours post–transfection of 2×10^6^ BHK–21 cells with 100 ng of the indicated RNA transcripts. The cells and supernatants were harvested at 72 hours post–transfection, and the number of genomic RNA molecules was calculated by quantitative real–time RT–PCR, as described in Materials and Methods (limit of detection 8 viral RNA molecules/cell, indicated as a dashed line). **E**, Viral titre (PFU/ml) in the transfected cultures (both the supernatant and cells subjected to freeze–thawing) described in D. Plaque assays were performed as described in Materials and Methods. Plaque development was permitted for 48h (limit of detection 5 PFU/ml, indicated as a dashed line). Measurements in D, E were carried out in triplicate and standard deviations are given.

Previous studies showed that FMDV encoding VPg_3_ as the only copy of VPg (but not FMDV encoding only VPg_1_ and VPg_2_) is infectious in cell culture [11], [12]. It was not clear whether lethality was due to absence of VPg_3_ or to absence of the proteolytic cleavage site between VPg_3_ and 3C [11]. To address whether VPg_3_ is essential for FMDV replication, and to investigate the function of repeated VPg genes in Aphthoviruses, we have designed FMDVs with only VPg_1_ (termed V1), only VPg_3_ (termed V3), and chimeric viruses containing most of VPg_1_ but a short C-terminal portion of VPg_3_ that restores the cleavage site between VPg_1_ and 3C. The chimeric–VPg viruses have been termed V19–4 (first 19 residues from VPg_1_ and last 4 residues from VPg_3_) and V15–9 (first 15 residues of VPg_1_ and last 9 residues from VPg_3_) (Figure 1). All mutant viruses, except V1, were competent in replication when the corresponding RNAs were transfected into BHK–21 cells, as evidenced by quantification of virus–specific RNA and proteins (Figure 2). The results show that a complete sequence of VPg_3_ is not essential for viral replication, but VPg_1_–containing mutants require additional residues from VPg_3_ in their C–terminus for replication.

Viral proteins, measured by metabolic labelling and Western blot analysis, were detected with all mutants except V1 (Figure 2A–C). As expected, all mutant constructs that expressed replication-competent RNAs gave rise to a P3 polyprotein precursor that displayed higher mobility than wild type (WT) P3, due to the deletion of two VPg copies (Figure 2B and 2C). The pattern of processed P3 proteins in the expression products of V19–4 and V15–9 supports an efficient processing of the precursor 3BC into 3B and 3C, in agreement with the introduction of a functional 3C cleavage site in the mutants (Figure 2C). Quantification of total (intracellular and extracellular) viral RNA collected from cell cultures transfected with mutant FMDVs yielded 10^4^–10^5^ viral RNA molecules/cell (vRNA/cell) for mutants V3, V15–9 and V19–4, a value which is 10– to 100–fold lower than that obtained for FMDV WT (Figure 2D). Mutant V1 showed a viral RNA level that was 10,000-fold lower than WT (<10^2^ viral RNA molecules/cell), suggesting that replication of V1 is either drastically reduced or abrogated (Figure 2). It cannot be excluded that the low viral RNA level detected after transfection with V1 RNA could be a remnant of the RNA used in the transfection (10^4^ viral RNA molecules/cell), and it confirms the positive replication of the other mutants. In summary, there is no requirement of a specific VPg sequence for FMDV viability since the three different VPg versions confer replication–competence to a similar extent. However, the presence of a 3C protease cleavage site between VPg and 3C seems to be needed for viral RNA synthesis.

### FMDVs expressing a single VPg lack plaque–forming capacity

Samples from the cell culture supernatants of BHK–21 cells transfected with V1, V3 or V19–4 RNAs did not include detectable viral infectivity in a standard FMDV plaque assay (<5 PFU/ml), with plaques visualised at 48 hours post–plating (FMDV WT plaques are readily detectable at 24 hours post–plating) (Figure 2E). Samples from transfection with V15–9 presented detectable infectivity [8(±3)×10^2^ PFU/ml], albeit 1,000- to 10,000-fold lower than WT [3(±2)×10^6^ PFU/ml]. The absence of plaque development with V3 or V19–4 contrasted with the large amounts of total viral genomic RNA detected in the same cell cultures (Fig 2D), and with the observed intracellular levels of viral proteins (Figure 2A–C). The specific infectivity (SI, ratio between viral titre and the number of viral RNA molecules) of viruses expressing only one VPg is lower than 1∶20,000,000, which is at least 500–fold lower than that of FMDV WT (1∶40,000). For standard FMDV C–S8c1, the ratio of infectious particles to total number of physical particles was previously estimated in 1∶7,000 to 1∶10,000 [38], and the ratio decreases when FMDV is subjected to mutagenesis [39]. Thus, viruses expressing a single VPg are competent for viral protein synthesis and RNA synthesis, but defective in plaque development (Figure 2).

### The specific infectivity of mutant FMDVs is gradually regained upon passage in BHK–21 cells

**Figure 3:**
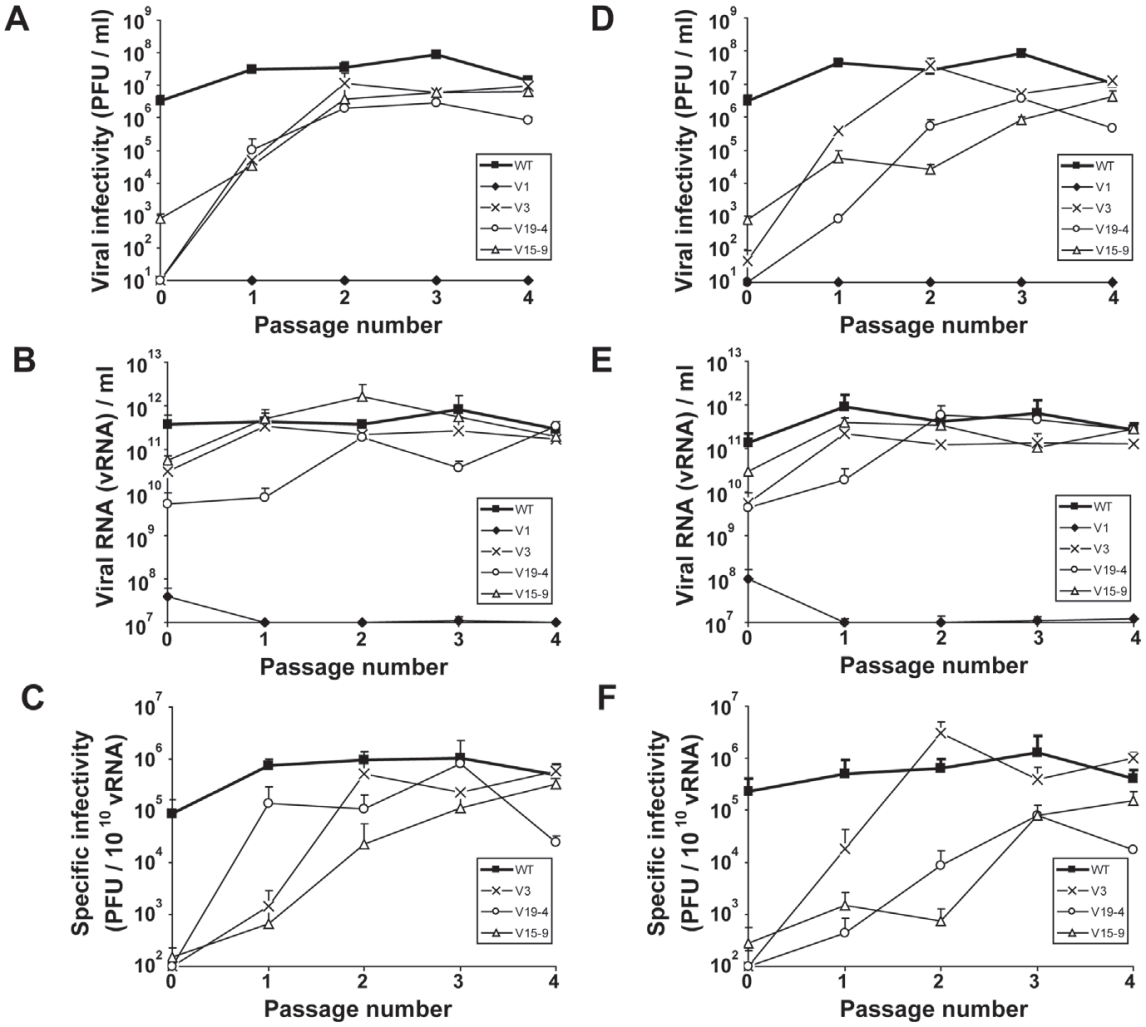
Recovery of infectivity of FMDV mutants expressing one VPg, upon serial passages in BHK–21 cells. 2×10^6^ BHK-21 cells were transfected with 100 ng (series *A*) (panels A, B, C) or 500 ng (series *B*) (panels D, E, F) of the FMDV transcripts indicated in the boxes. Cells and cell culture supernatants were collected at 72 h post-transfection (passage 0). Successive passages were carried out by infecting BHK–21 cells with the viruses from the cell culture supernatant of the previous passage, and samples were collected when cytopathology was complete (generally 20 to 30 hours pi). **A**, **D**, Viral infectivity as a function of passage number; plaque development was for 48 hours. **B**, **E**, Number of genomic RNA molecules in the cell culture supernatants. **C**, **F**, Specific infectivity calculated as the number of PFU per 10^10^ viral RNA genomes, from the data given in A, B and D, E. Measurements were carried out in triplicate and standard deviations are given. Procedures for titration of infectivity and determination of viral RNA levels are described in Materials and Methods.

To investigate whether the capacity of FMDV mutants V1, V3, V19–4 and V15–9 to form plaques could be restored upon further virus replication, the virus present in supernatants collected from cell cultures initially transfected with 100 ng (series A) or 500 ng (series B) of V1, V3, V19–4 and V15–9 RNA was serially passaged in BHK–21 cells. At the first passage (p1), cytopathology was complete in cells infected with V3 and V15–9, but affected only 10% of the cells infected with V19–4, at 70–90 hours post–infection (hpi), and no cytopathology was observed with the supernatants that should contain the progeny of V1 RNA. For FMDV WT, complete cytopathology was observed at 30 hpi, in agreement with previous observations [40]. Infectivity was detected for all viruses (except for V1) at p1, although mutant viruses yielded lower titres and SI values than WT. Successive passages led gradually to a more extensive cytopathology at shorter times pi, and to increases in viral titre and SI for all mutants tested, except for V1 (Figure 3). Plaque development by FMDV on BHK–21 cell monolayers is a reflection of cell detachment as a manifestation of virus infection. Therefore, in the assays described here, cytopathology refers as cell rounding and detachment as a result of the cellular modifications previously described for BHK–21 cells lytically infected by FMDV [36], [37].

### Multiple genomic sites are involved in recovery of the plaque–forming phenotype

To investigate whether plaque–forming capacity was regained via adaptive mutations in the viral genome, the entire genomic consensus sequence [except the genomic 5′ and 3′ termini, and the nucleotides upstream and downstream from of the polyC tract] of six mutant populations at passage 2 (series A and B for mutants V3, V19–4 and V15–9) was determined. Although no mutation was repeated in all populations, amino acid substitution R55W in non–structural protein 2C was present in populations V3B, V19–4B and V15–9B (Table 1). This result suggested that substitution R55W in 2C could be involved in restoring the plaque–forming phenotype in these three populations. The only substitution that affected VPg was E8K found in population V19–4A (Table 1).

**Table 1:** Substitutions found in FMDV mutants that encode one VPg, upon passage in BHK–21 cells a.

Genomic region b	Mutation c	Amino acid substitution c	Presence in biological clones from V3A at passage 1 d	Presence in uncloned population at passage 2 d
*L*	A1244G	D69G	—	V19–4A
	A1292U	Q85L	—	V3B
*2C*	C4507U e	R55W e	c1, c4 e	V19–4B; V15–9B; V3B e
	C4778U	T135I	c2	—
	A4988G	K215R	—	V15–9B
*3A*	A5422C	I42L	c3, c5	—
	A5609C	Q104P	c4	—
*VPg_1_-coding region*	G5779A	E8K	—	V19–4A
*3C*	C6060U	—	c1	—
*3D^pol^*	A7487G	E293G	c1	—

a The consensus sequence of the entire viral genome was determined for FMDV populations V3, V19–4 and V15–9 (series A and B) after 2 passages in cell culture (experiment described in Figure 3). The sequence of viral genomic regions 2C, 3A, 3B (VPg), 3C and 3D (residues from genomic position 4200 to 8115) was determined for 5 independent biological clones isolated from population V3A at passage 1. The origin of each population is described in the text and in Figure 3.

b FMDV genomic region analysed [15].

c Mutations and deduced amino acid substitutions are relative to the sequence of the parental clone FMDV WT (pMT28, described in Materials and Methods); residue numbering is according to [49]. Amino acid residues (single–letter code) are numbered individually for each protein from the N– to the C–terminus. Procedures for nucleotide sequencing and identification of FMDV genomic regions are described in Materials and Methods.

d Populations and biological clones in which the substitutions indicated in ^c^ were found.

e Mutation C4507U (that corresponds to amino acid substitution R55W in 2C) was repeatedly found in 3 out of 6 mutant FMDV populations at passage 2, and 2 out of 5 biological clones from population V3A at passage 1 (underlined).

Recovery of the plaque–forming phenotype was not associated with any dominant mutation in two out of six populations at passage 2 (V3A and V15–9A). To determine whether the mutant spectrum of V3A included genomes with the same mutations found in the other lineages, the P2–P3–coding region of virus isolated from 5 individual plaques derived from population V3A at passage 1 was sequenced. Each clone included non–synonymous mutations in 2C and/or 3A, and two clones presented replacement R55W in 2C (Table 1). The results suggest that plaque–development in the VPg mutants can be attained by any of a number of substitutions in non–structural proteins 2C, 3A and/or VPg. Since R55W in 2C was found in several clones and populations, its effect on FMDV progeny production and cytopathology was further investigated.

### 2C substitution R55W increases the specific infectivity of FMDV expressing a single VPg

**Figure 4:**
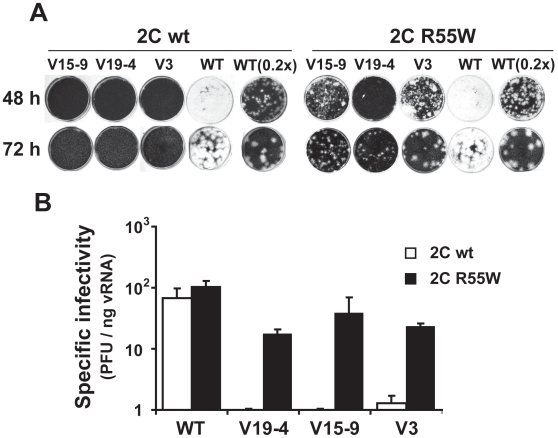
Restoration of the plaque–forming phenotype in FMDV mutants by replacement R55W in 2C. RNA transcripts (10 ng for 48 hours of plaque development and 1 ng for 72 hours plaque development) were transfected into 2×10^6^ BHK–21 cells. At 2 hours post–transfection cell culture supernatants were removed and the cells were overlaid with semisolid agar medium. **A**, Plaque assay of directly transfected FMDV RNA transcripts encoding either wild type 2C (left) or 2C with substitution R55W (right). For mutants V15–9, V19–4 and V3, plaques were detected when 2C included substitution R55W. WT(0.2x) represents transfections with a fifth of the amount used for the rest of the assays. Plaques for mutant V19–4 (R55W) were not detected at 48 hours post–transfection. V3 formed some plaques at 72 hours post–transfection but not at 48 hours post–transfection. The number of plaques formed by V3 was 18–fold lower than the number formed by V3 encoding R55W in 2C. **B**, Specific infectivity measured as the ratio of the number of PFU at 72 hours post–transfection to the amount of viral transcript used in the electroporation. Assays were carried out in triplicate, and standard deviations are given. Procedures for plaque assays of transfected cells are described in Materials and Methods.

To determine whether substitution R55W in 2C could restore the plaque–forming capacity of FMDV mutants with alterations in 3B (FMDV mutants V3, V19–4 and V15–9, Figure 1), the corresponding mutation was introduced in each of the mutant constructs that express one VPg. All mutants regained the plaque–forming phenotype (Figure 4A) that resulted in 17– to 190–fold increase of specific infectivity of each viral RNA transcript (Figure 4B). To minimize the emergence of adaptive mutations during plaque development, serially diluted viral RNA transcripts were transfected into cells, and plaque development was permitted under semisolid agar medium. Plaques were formed by all mutants expressing only one VPg and 2C with R55W, and they were visible at 3 days post–transfection. No plaques were formed by their RNA counterparts expressing wild type 2C, under the same conditions. By 2 days post–transfection, plaques were already detected for V15–9 and V3 with R55W in 2C, but not for V19–4 with R55W in 2C (Figure 4). Thus, 2C substitution R55W is sufficient to restore the plaque–forming capacity of the mutants tested that express one VPg.

### R55W in 2C increases cytopathic effect and results in enhanced viral release from cells

**Figure 5:**
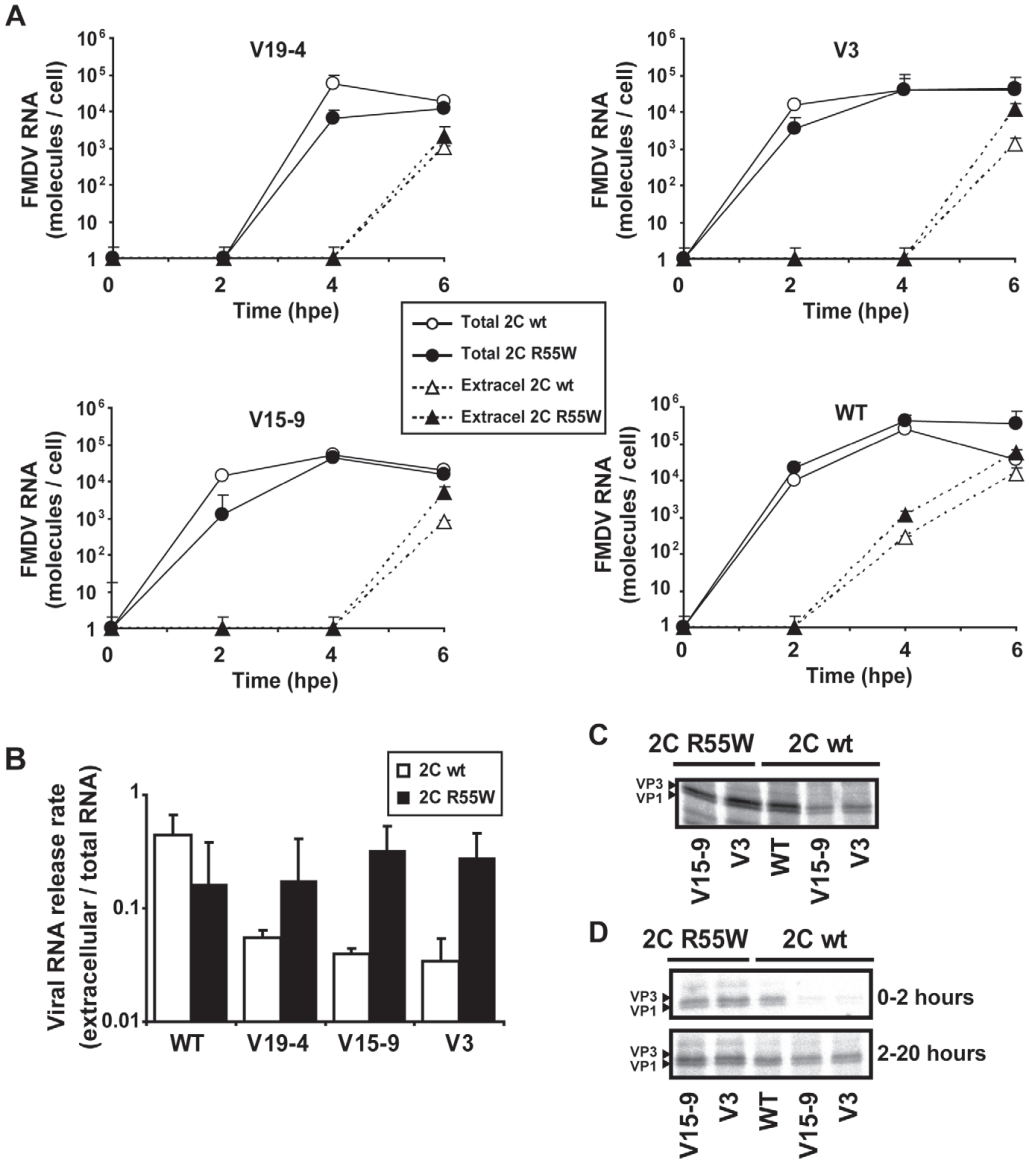
Effect of replacement R55W in 2C in the release of FMDV RNA from cells. 10^6^ BHK–21 cells were electroporated with 8 µg of either V19–4, V3, V15–9 or WT RNAs. Then the cells were transferred to M24–wells. **A**, Total viral RNA (intracellular and extracellular) and extracellular RNA were quantified by real–time RT–PCR, at the indicated hours post-electroporation (hpe). The amount of FMDV RNA was normalised to the number of cells seeded in the corresponding wells. The viral RNA (total and extracellular) measured just after electroporation was subtracted from each corresponding value. Note that RNAs encoding R55W in 2C are significantly more efficient in promoting release of viral RNA from cells at 6 hours post–transfection than those expressing wild type 2C (p<0.05; paired *t*–Test). **B**, Ratio of FMDV RNA released from the cells (ratio of extracellular viral RNA to total RNA) at 6 hours post–electroporation, determined from the data shown in A. Assays in A and B were carried out in triplicate and standard deviations are given. **C**, Detection of capsid proteins VP3 and VP1 in mutant FMDVs expressing 2C with or without replacement R55W. 10^6^ BHK–21 cells were electroporated with 8 µg of viral RNA. At 3 hours post–electroporation, supernatants were removed and DMEM without Met and Cys, but supplemented with [^35^S] Met–Cys, was added. At 4 hours post–electroporation, supernatants were removed and cells were collected to analyse the synthesis of the viral capsid proteins VP3 and VP1. **D**, Extracellular samples of cell cultures electroporated with the indicated mutant FMDV RNAs, labelled as described in C, were collected at 2 (0-2 hours) and 20 (2-24 hours) hours post–labelling (6 and 24 hours post–electroporation). Samples were analysed by SDS–PAGE (15% acrylamide). VP3 and VP1 were detected at early time (4 to 6 hours post–transfection) in the supernatant of cells transfected with 8 µg of V15–9 or V3 encoding 2C with R55W, but not with the same amount of RNA from constructs encoding wild type 2C. At later times (6 to 24 hours post–transfection), VP3 and VP1 were detected in the supernatant of cells transfected with any of the constructs tested. Procedures are further detailed in Materials and Methods.

To investigate the effect of R55W 2C in FMDV replication, viral RNA and viral protein levels of FMDVs expressing 2C with either R55 (wild type) or W55 (adaptive substitution) were quantified at different times post–electroporation. Positive RNA replication was observed for all mutants (V3, V19–4 and V15–9 with or without R55W in 2C) with RNA levels reaching an amount at least 10-fold higher than levels quantified at the time of electroporation (Figure 5A). To ascertain the presence of newly synthesised viral RNA molecules, the background RNA value scored at the time of the electroporation was subtracted from each of the values obtained at different times after electroporation. The results (Figure 5A) indicate that the presence of R55W in 2C of the FMDV mutants increased the amount of extracellular viral RNA without any significant increase in the total viral RNA levels (intracellular plus extracellular). The ratio of extracellular to total viral RNA molecules was estimated to be in the range of 0.1 to 0.4 for mutants encoding R55W in 2C, and 0.03 to 0.04 for mutants encoding wild type 2C (Figure 5B).

The selective increase of extracellular RNA suggests increased exit of viral particles. This implies that higher extracellular concentrations of capsid proteins should be found for mutants expressing 2C with R55W than for the same mutants expressing wild type 2C. This was confirmed by determining extracellular levels of capsid proteins VP1 and VP3. First, the synthesis of capsid proteins VP1 and VP3 was ascertained in the electroporated cells by pulse-labeling with [^35^S] Met-Cys at 3 h to 4 h post-electroporation. The result (Figure 5C) revealed the presence of VP1 and VP3 in the cells infected with mutants V15-9 and V3, with or without R55W in 2C (excluding the cell culture supernatant for the analysis). In contrast, when the extracellular levels of VP1 and VP3 were examined at early times post-electroporation, they were barely detectable for the mutants that expressed 2C with R55 (Figure 5D). The difference tended to diminish when the pulse-labeling was extended for several hours post-electroporation (Figure 5D).

**Figure 6:**
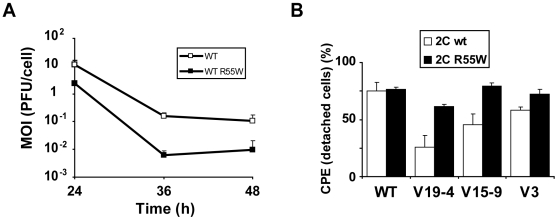
Effect of replacement R55W in 2C on FMDV cytopathology. **A**, BHK-21 cell-killing assay [40] carried out with either FMDV WT (expressing three VPgs) with the standard 2C (abbreviated as WT) or FMDV WT with substitution R55W in 2C (abbreviated as WT R55W). Serial dilutions of either FMDV WT or FMDV R55W were applied to wells containing 10^4^ BHK-21 cells each. The plot depicts the minimum MOI (PFU/cell) required to cause complete cytopathology (detachment of all cells from the monolayer) at the indicated hours post-infection, as previously described [40]. Results are the average of four independent determinations. Note that the presence of R55W in 2C decreases the MOI needed to cause complete cytopathology for a given time. **B**, Quantification of cytopathology (percentage of cells detached from the monolayer) in cells with RNA from wild type (WT) or mutants V19-4, V15-9 or V3 expressing either wild type 2C or 2C with substitution R55W. At 8 h post-electroporation the total number of cells and the number of detached cells were counted; a correction was introduced to account for the number of cells that were detached due to the electroporation by parallel measurements with mock-electroporated cells (around 50% of cell detachment). Results are the average of three determinations.

A standard cell killing assay previously developed for FMDV in BHK-21 cells [40], [41] could not be directly applied to measure the effect of substitution R55W in 2C to mutants V19-4, V15-9 and V3 because infectious (cell-detaching) particles could not be produced in sufficient amounts. However, the cell killing assay was applied to wild type FMDV expressing either wild type 2C or 2C with R55W and the results showed 8- to 20-fold increases of cytopathology associated with substitution R55W in 2C (Figure 6A).

In other assays, the number of detached cells versus the total number of cells was measured following electroporation with V3, V19–4 and V15–9 RNAs, expressing either 2C wild type or 2C with R55W, as a means of circumventing the requirement of infectious virus particles. The results (Figure 6B) show that the proportion of cells detached was 62±3%, 79±6% and 72±10% in transfections with mutants V19–4, V15–9 and V3 encoding R55W in 2C, respectively, while these values were 26±11%, 45±10% and 58±3% in transfections with the same mutants encoding the wild type 2C, and this difference was statistically significant (p = 0.0002; Student's *t*–Test). To prevent multiple–step growth conditions that may have affected the interpretation of results, the experiment was carried out by using 5 µg of viral RNA for 10^6^ BHK-21 cells, a sufficient amount to ensure that more than 90% of the cells were efficiently transfected Therefore, substitution R55W in 2C favors both release of viral particles and cytopathology.

### Further analyses with FMDV mutants expressing one VPg

The major defect of FMDV mutants V19-4, V15-9 and V3 that express a single VPg is the absence of cytopathology associated with limited release from cells with no impairment of intracellular viral RNA levels. To test whether other viral functions might also be affected in these mutants, cell entry and intracellular viral protein synthesis were also examined.

To determine whether mutants expressing a single VPg could also manifest a defect in cell entry, the capacity of mutants V19-4 and V3 expressing either wild type 2C or 2C with R55W to enter cells was determined. For this purpose, viral samples were collected from cell cultures at 4 hours post–transfection by freeze-thawing the cells to liberate virus, and then applied to a BHK–21 cell monolayer. The input FMDV RNA applied to the monolayer and the FMDV RNA that entered BHK-21 cells were quantitated by real time RT-PCR. The proportion of viral RNA released from transfected cells that entered new cells was 3.0±0.3% for V19-4 and 3.5±2.0% for V3 that expressed wild type 2C; the corresponding values were 2.6±1.3% and 3.0±1.2% for the same mutants that expressed 2C with R55W. These data suggest that defects in plaque-forming capacity observed are not due to a defect in viral entry once the mutant FMDvs have been released from the cells.

**Figure 7:**
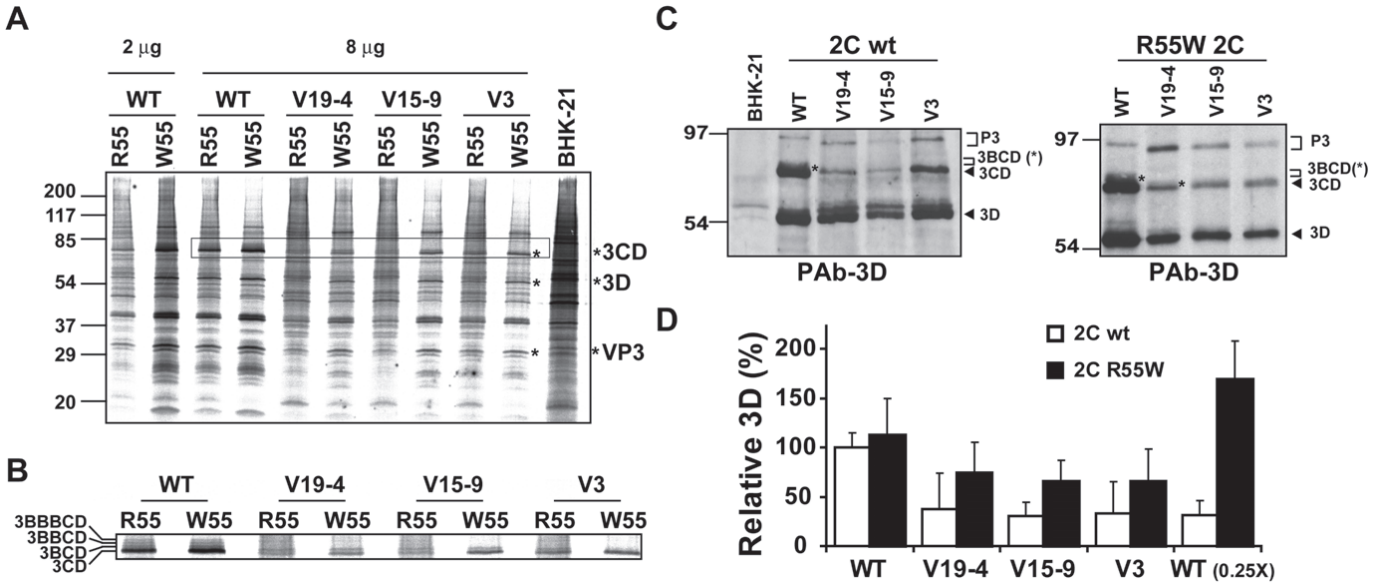
Viral proteins expressed by mutant FMDVs encoding either wild type 2C or 2C with R55W. **A**, Electrophoretic analysis of proteins labelled with [^35^S] Met–Cys. 10^6^ BHK–21 cells were electroporated with 2 or 8 µg of the indicated viral RNAs; At 3 hours post–electroporation, supernatants were removed and DMEM without Met and Cys but supplemented with [^35^S] Met–Cys was added. One hour later, cell culture supernatants were removed and cells were collected in loading buffer, proteins were resolved in SDS–PAGE (15% acrylamide), and the gel was dried and exposed. Viral proteins, identified by their reactivity with specific MAbs [62], are indicated with an asterisk. **B**, Close-up view of the bands in A corresponding to 3CD and 3BCD precursors. Wild type FMDVs expressing 3 copies of VPg show three precursors of lower mobility than 3CD that may correspond to 3BCD, 3BBCD and 3BBBCD, respectively. Mutant FMDVs expressing one VPg show only one precursor above 3CD that may correspond to 3BCD. **C**, Western blot analysis of samples described in A. Proteins were visualised with a specific polyclonal antibody against FMDV 3D [62]. Molecular size markers were run in parallel and their position is indicated on the left. The positions of viral proteins P3, 3CD, and 3D are indicated [62]. Note that precursor P3 (3ABCD) from the mutant transcripts expressing one VPg displayed a higher mobility than WT P3. A band above 3CD was detected in some lanes (indicated with an asterisk) that may correspond to precursor 3BCD in V19–4 R55W and to precursors 3BCD, 3BBCD and/or 3BBBCD in FMDV WT, with or without R55W in 2C. **D**, Proportion of viral polymerase 3D (as percentage of the value obtained in the electroporation with 8 µg of FMDV WT transcript), measured by densitometry of electropherograms as that shown in A. Values are the average of triplicate protein analyses of independent labelling experiments, and standard deviations are given. WT (0.25x) indicates transfections with 2 µg of WT. The assignment of viral proteins was based on reactivity with specific monoclonal antibodies [39], [62], [66]. Procedures are described in Materials and Methods.

To compare intracellular protein synthesis directed by the mutant FMDVs, proteins were metabolically labeled between 3 and 4 hours post–electroporation. BHK–21 cells infected with mutants V3, V19–4 and V15–9 encoding R55W in 2C presented modestly higher levels of viral proteins than the same mutants expressing a wild type 2C (Figure 7). Therefore, a limitation in protein synthesis cannot be related to a defect in viral release from cells.

### Substitution E8K in VPg contributes also to increased viral release in mutant viruses expressing one VPg

Mutant V19–4 (R55W) showed a delayed plaque formation relative to other mutants encoding R55W in 2C (Figure 4). To investigate whether additional replacements could accelerate plaque formation in this virus, populations V19–4A and V19–4B at passage 2 (depicted in Table 1 and Figure 3) were subjected to 25 additional passages in BHK–21 cells. E8K in VPg_1_ was the only replacement repeatedly found in the two V19–4 lineages at passage 27 (Table 2). Interestingly, R55W in 2C became dominant during the 25 additional passages in lineage V19–4A in which R55W was not present at passage 2 (compare Tables 1 and 2). However, in lineage V19–4B W55 was dominant at passage 2 but it had reverted to R55 by passage 27. This reversion was accompanied by dominance of substitution I85V in 2C (Table 2). Other amino acid replacements were identified in other viral proteins of passage series A and B (Table 2).

**Table 2:** Mutations in populations V19–4A and V19–4AB after 27 passages in BHK–21 cells a.

Genomic region b	Mutation c	Amino acid substitution c	V19–4 population d
*L*	U1103C	L22P	V19–4B
	A1244G	D69G	V19–4A
*VP1*	A3649G	T148A	V19–4B
	C3650C/U	T148M	V19–4A
	C3653A/C	T149K	V19–4A
*2C*	C4507U	R55W	V19–4A
	*U4507C* e	*reversion to R55* e	*V19–4B* e
	A4597G	I85V	V19–4B
*3A*	A5573G	E92G	V19–4B
*VPg_1_-coding region*	G5779A f	E8K f	V19–4A; V19–4B f
*3C*	U6534A	—	V19–4B
*3D*	U7341C/U	—	V19–4A

a The consensus sequence of the entire viral genome was determined for FMDV populations V19–4A and V19–4B after 27 passages in cell culture.

b FMDV genomic region analysed [15].

c Mutations and deduced amino acid substitutions are relative to the sequence of the parental clone FMDV WT (pMT28, described in Materials and Methods); residue numbering is according to [49]. Amino acid residues (single–letter code) are numbered individually for each protein from the N– to the C–terminus. Two residues separated by a bar indicate a mixture of two nucleotides in the population, according to the sequence chromatogram pattern. Procedures for nucleotide sequencing and identification of FMDV genomic regions are described in Materials and Methods.

d Populations in which the substitutions indicated in ^c^ were found.

e Mutation C4507U (that corresponds to amino acid substitution R55W in 2C) was dominant in V19–4B at passage 2 (Table 1) and had reverted by passage 27 (highlighted in italics).

f Substitution E8K (G5779A) was found repeated in population V19–4 at passage 27 in series A and B (underlined).

**Figure 8:**
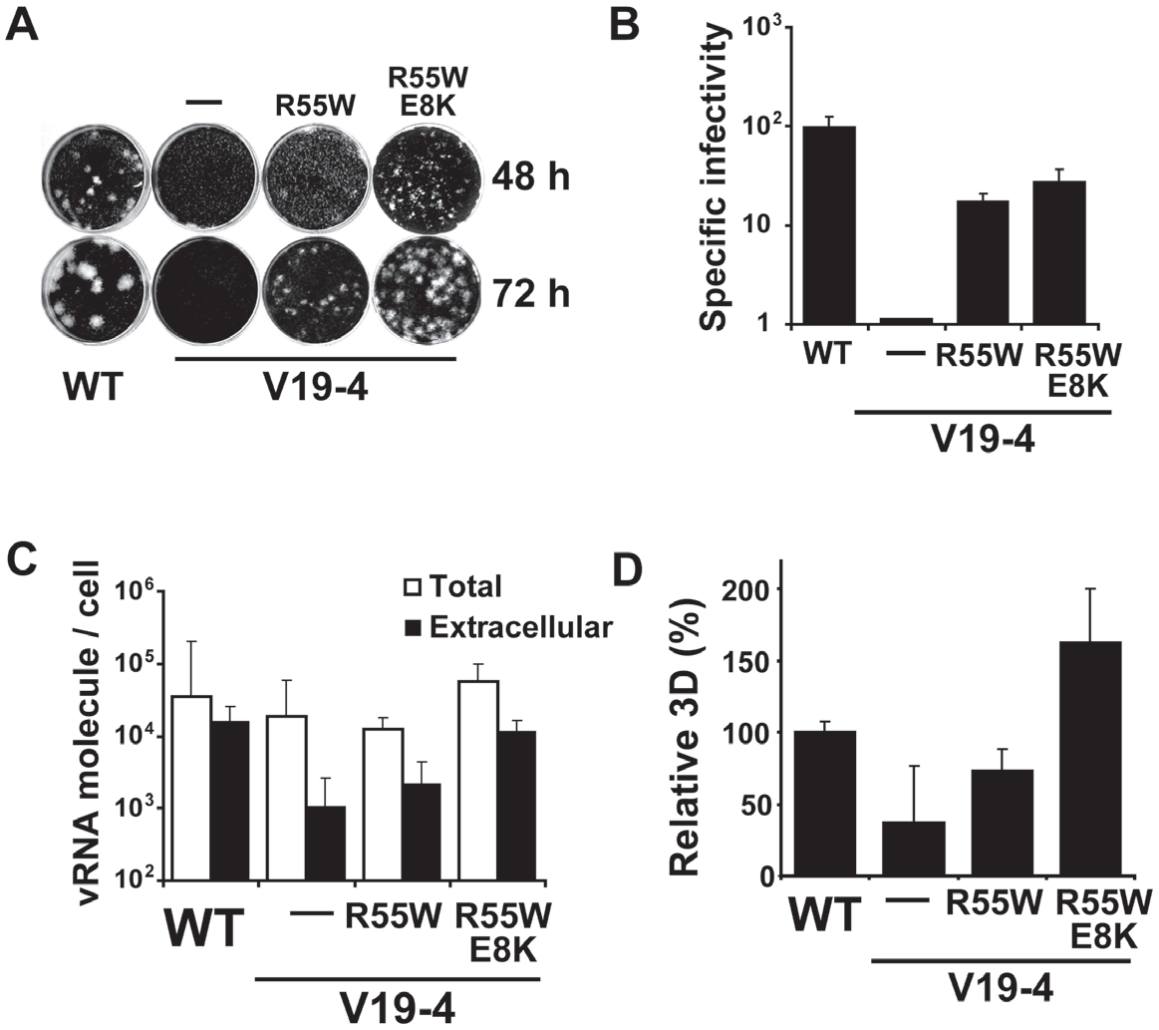
Effect of replacement E8K in VPg on the specific infectivity of V19–4 (R55W). **A**, Plaque assay with cells transfected with RNA transcripts of WT or the indicated mutant constructs. At 2 hours post–transfection, the cell culture supernatants were removed and the cells were covered with semisolid agar. Plaques were visualized either at 48 or 72 hours post–transfection. Note that substitutions R55W in 2C and E8K in VPg enhanced the plaque-forming capacity of V19–4. **B**, Specific infectivity (calculated as the ratio of the number of PFUs obtained to the ng of RNA used in the electroporation) for WT and the indicated V19–4 mutants. **C**, Total and extracellular FMDV RNAs measured at 6 hours after electroporation of BHK–21 cells with WT and the indicated V19–4 mutant RNAs. **D**, Amount of viral polymerase (3D) determined by metabolic labelling after electroporation of BHK–21 cells with the indicated RNAs, and densitometry of the relevant ptotein band. The results are expressed as percentage of the amount measured following electroporation with WT RNA, which is taken as 100%. Values in B, C, D are the average of 3 determinations; standard deviations are given. Procedures are described in Materials and Methods.

Since R55W in 2C and E8K in VPg_1_ were found together in V19–4A at passage 27, the effect of both substitutions together in the same genome in the sequence context of V19–4 was investigated. The double mutation restored plaque–forming capacity, with minute plaques detected at 48 h post–transfection (Figure 8A). The specific infectivity of V19-4 increased 10- to 100-fold as a result of expressing 2C with R55W alone or together with VPg with E8K (Figure 8B). The extracellular viral RNA was 5– to 11–fold higher for V19–4 (R55W,E8K) than for either V19–4 or V19–4 (R55W) (Figure 8C), and this difference was statistically significant (P<0.0001; unpaired *t*–Test). Metabolic labelling of cells transfected with V19–4 (R55W,E8K) RNA revealed that viral protein synthesis was increased 2.2– to 4.4–fold relative to V19–4 or V19–4 (R55W). Protein levels were 1.6–fold higher for V19–4 (R55W,E8K) than for wild type FMDV (Figure 8C). These measurements suggest that viral release into the extracellular medium was enhanced by substitution E8K in VPg.

In summary, FMDV expressing only one VPg is defective in cell–to–cell propagation and this defect is reverted by an increase in viral release, concomitantly with restoration of cytopathology, mediated by substitutions in 2C, VPg or other non-structural proteins.

## Discussion

Two features of RNA virus evolution are relevant to the interpretation of the results with VPg mutants of FMDV reported here. One is the multifunctional nature of many (probably most) proteins encoded in the compact RNA genomes, particularly in the case of picornaviruses in which several intermediates obtained in the processing of the polyprotein play essential roles in the virus life cycle. In the present study, multifunctionality was manifested by the participation of 2C in the compensation of a defect in the release from cells of FMDVs that do not express the 3 VPg versions encoded by wild type FMDV [42]. Such compensation suggests a role of 2C in functions additional to those described for this non–structural protein [20], [21], [24]-[27], [43]. The second relevant feature of RNA virus evolution is the functional connection among different genomic regions, in this case between VPg and other non–structural proteins, regarding expression of phenotypic traits [44].

The results reported here indicate that the replication-competent FMDV mutants that expressed a single VPg were deficient in plaque formation, a defect related to the inability to cause cytopathology. Cell rounding and detachment from the monolayer precede cell death. The mechanism (apoptosis, necrosis, autophagy) by which FMDV kills BHK-21 cells in cell culture has not been elucidated [45], and our attempts to confirm or exclude apoptosis of BHK-21 cells following infection by FMDV have not been conclusive (C. Perales, unpublished observations). We have observed that the first step towards cell death –as measured either by trypan blue staining or by fluorescence-activated cell sorting (FACS) using propidium iodide that gave equivalent quantifications [46]– is cell rounding and detachment of the monolayer. The capacity to produce cytopathology was rapidly restored upon passage of the mutant viruses in BHK-21 cells, that acquired amino acid substitutions in non-structural proteins, including R55W in 2C. The results suggest that substitution R55W in 2C permitted FMDVs that expressed a single VPg to attain a critical number of viral particles released from cells, that are associated with cytopathology. An approximate value of 5 infectious particles released per BHK–21 cell was estimated as the minimum number needed to develop a plaque under our experimental conditions (Table 3). This is the number of particles released per cell estimated for mutant V19–4 (R55W) which is near the threshold of not being competent in plaque development; the plaques formed by this virus were visible only at late times post–transfection. The functional basis for this critical number is not known.

**Table 3:** Relative amounts of mutant FMDV RNA released from cells a.

Electroporated FMDV transcript b	Viral RNA released (%) c	Calculated number of infectious particles released per cell d	Plaque-forming capacity e
**WT**	100±48	34±30	Yes
**WT R55W**	356±65	121	Yes
**V19–4**	7±1	2	No
**V19–4 R55W**	14±10	5	At 72 hpt
**V15–9**	5±1	2	No
**V15–9 R55W**	32±15	11	Yes
**V3**	9±5	3	No
**V3 R55W**	79±30	27	Yes

a 10^6^ BHK–21 cells were electroporated with 8 µg of FMDV RNA, and the amount of extracellular FMDV RNA at 6 hours post–electroporation was quantitated as detailed in Materials and Methods.

b Mutant FMDVs analysed. Their construction is described in Materials and Methods, and depicted in Figure 1; R55W indicates the presence of replacement R55W in 2C.

c Viral RNA released per cell for the indicated transcripts. Values are expressed as the percentage of the amount of RNA released per cell in the transfections with FMDV WT. Each value is the average of at least three determinations; standard deviations are given. Relative viral RNA released per cell in mutant FMDVs defective in plaque–forming capacity is below 20% of FMDV WT (calculated from data shown in Figure 5).

d Calculated number of infectious particles released per cell for wild type and mutant FMDVs. An average number of 34 PFU are released per cell in infections with FMDV WT (data in Figure 3). The calculation assumes that one FMDV WT infectious particle is equivalent to one PFU, and that the number of infectious particles released by each mutant virus correlates with the viral RNA released.

e Plaque–forming capacity of mutant FMDV transcripts (data in Figure 4). Yes indicates the ability of mutant transcripts to develop plaques in 48 h. No indicates that no plaques were observed at 72 hours post–electroporation. Plaque development with V19–4 R55W was only observed at 72 hours post–electroporation. Procedures for plaque assays of viral transcripts are detailed in Materials and Methods.

Residue 55 of 2C [and also residue 85, substituted in the R55W revertant of V19–4B (see Results)] is located in the region comprised between the expected membrane-binding domain and the predicted NTPase/helicase domain A, and it is a variable residue when 2Cs of different picornaviruses are aligned. Positions 55 and 85 have not been identified as related to resistance to guanidine in FMDV [47], [48] and therefore, from the data presently available no connection between 2C positions 55 and 85 and FMDV replication is apparent. It cannot be excluded that the established role of 2C in cellular membrane rearrangements during picornaviral infection [27]–[31] might be connected with its effect in virus release and cytopathology.

With the assumption that cytopathology by FMDV precedes cell death, several determinants of BHK–21 cell killing were previously mapped in the IRES, the viral capsid, and non–structural proteins of the virus [40], [49], [50]. A lack of correlation between replicative fitness and BHK–21 cell killing capacity was unveiled by analysis of the behavior of chimeric FMDVs. While fitness determinants were scattered along the genome, cell killing determinants were concentrated in several specific genomic regions, including 2C [40]. Three substitutions in 2C (S80N, T256A and Q263H) enhanced BHK–21 cell killing. Since these substitutions are distant from substitution R55W that multiple sites in 2C are related to FMDV cytopathology.

The molecular basis of the increased cytopathology observed for mutants that encode 2C with substitution R55W remains unknown. Since a modest increase in viral protein synthesis in these mutants is observed despite no increase in viral RNA levels, an appealing hypothesis is that 2C has some involved in translation, and that R55W might enhance protein synthesis modestly but to a level sufficient to contribute to enhanced cytopathology. The effect of substitution R55W could be exerted during polyprotein processing, by rendering 2C more sensitive to polyprotein cleavage mediated by 3C. Distance effects of amino acid substitutions on FMDV poplyprotein processing have been previously documented [51]. Other interpretations are possible. For example, since 2C is a multifunctional protein [20]–[31], substitution R55W may alter virus-host interactions that lead to enhanced cytopathology, being the slight increase of protein synthesis an indirect consequence of alterations in other viral functions.

Substitution E8K was also found associated with the one-VPg mutants that regained capacity to cause cytopathology. The crystal structure of the complex between 3D and VPg predicts that E8 participates directly in the interaction with the viral polymerase. Although E8 is far from the UMP residue bound to VPg (covalent bond Tyr3-UMP), substitution E8A has been shown to affect drastically the uridylylation of VPg by 3D [52]. The molecular basis of the phenotypic effect of VPg replacement E8K remains unknown.

Despite the fact that the behavior of the one-VPg FMDV mutants studied here suggests a functional connection between 2C and VPg in FMDV, to our knowledge a direct interaction between the two proteins has not been reported in picornaviruses. Nevertheless, PV 2C interacts with the VPg precursor 3AB (3B) [33]. In an FMDV–infected cell, a possible 3AB–2C interaction could be mediated by more than one VPg copy, and the alteration in the number of VPg copies or their amino acid sequence could perturb these 2C–3AB interactions. In addition to the replacements in 2C and 3B, substitutions in 3A were also found in populations and biological clones that regained the plaque–forming phenotype upon passage in BHK–21 cells, supporting that recovery of this phenotype can be mediated by any of a number of readjustments in the interaction among several non–structural proteins (Tables 1 and 2).

The present investigation has established non–structural proteins 2C, 3A and VPg as key determinants for modulating cytopathology in cell culture. For several picornaviruses it has been shown that replacements either in 2C or 3A can be associated with modifications of virulence, cell tropism or host range [40], [53]–[57]. Altogether, these data highlight the role of non–structural proteins in the adaptability to changing environments during picornavirus infections, with clear implications for viral pathogenesis.

## Materials and Methods

### Plasmids, cells and viruses

pMT28 is a pGEM–1 plasmid (Promega) that contains the complete cDNA genomic sequence of FMDV serotype C (FMDV WT: C-S8c1 as described in [41], [58], [59]). To produce infectious transcripts, pMT28 was linearised with *Nde*I (NEB), and transcribed with SP6 polymerase (Promega), following described procedures [60], [61]. Mutant plasmids encoding only one VPg copy were constructed by mutagenic PCR with primers harboring the desired deletion (overlapping upstream and downstream sequences of the region to be deleted). Two amplifications were made and then the DNA products were shuffled to introduce the deletion. The first amplification was performed with a forward primer spanning residues 3988 to 4009 and a mutagenic reverse primer spanning either residues 5980–5971 and 5826–5804 (to obtain the deletion of 5827 to 5970, mutant V1), or residues 5910–5899 and 5757–5735 (deletion of 5758–5898, mutant V3), or residues 5977–5959 and 5814–5792 (deletion of 5815–5958, mutant V19–4), or residues 5955–5944 and 5802–5781 (deletion of 5803–5943, mutant V15–9). The second amplification was performed with an antisense primer spanning either residues 7160 to 7141 and a mutagenic forward primer spanning residues 5816–5826 and 5971–5992 (to obtain the deletion of 5827 to 5970, mutant V1), or residues 5746–5757 and 5899–5912 (deletion of 5758–5898, mutant V3), or residues 5804–5814 and 5959–5992 (deletion of 5815–5958, mutant V19–4), or residues 5782–5802 and 5944–5958 (deletion of 5803–5943, mutant V15–9). Each pair of amplification products was shuffled by PCR amplification with internal primers spanning residues 4189 to 4211 (sense) and 7019 to 6997 (antisense). The mutagenised PCR products were inserted into pMT28, previously digested with *Bgl*II and *Cla*I (located at positions 4199 and 7003, respectively), by recombination using the *In–Fusion Dry and Down Mix* kit (Clontech).

For the construction of mutants encoding R55W in 2C (that correspond to mutation C4507U) two additional cloning sites, *Not*I (position 5412) and *Nsi*I (position 6393), were introduced into pMT28, leading to plasmid pMT30. We designed mutagenic primers that introduced substitutions C5412G and C5418G (*Not*I site) and G6393A (*Nsi*I site), without affecting the coding sequence. To obtain plasmid FMDV WT (R55W), the resulting pMT30 plasmid was digested with *Bgl*II (position 4199) and *Not*I (position 5412). RT–PCR amplification of RNA from population V19–4A at passage 27, that encodes 2C with R55W, was performed with primers spanning residues 4189 to 4211 (sense) and 5432 to 5392 (antisense). The amplification product was introduced into plasmid pMT30 by recombination using the *In*–*Fusion Dry and Down Mix* kit (Clontech).

Mutant FMDVs encoding one VPg and 2C with R55W were constructed by amplification of the mutant plasmids described above (V3, V19–4, V15–9) with primers spanning residues 5403 to 5442 (sense) and 6412 to 6374 (antisense). PCR amplification products were inserted into plasmid FMDV WT (R55W) previously digested with *Not*I and *Nsi*I (positions 5412 and 6393, respectively) by recombination with *In*–*Fusion Dry and Down Mix* kit (Clontech).

### Transfection of viral RNA transcripts into BHK-21 cells

Viral replication of each mutant FMDV was analysed by transfection of the corresponding transcript into BHK-21 cells. For this purpose we used both lipofection and electroporation methods. Lipofection was used to obtain mutant viral samples that were then passaged on BHK–21 cell monolayers, and also in plaque assays of viral transcripts. Electroporation was used to detect intracellular FMDV protein synthesis, and in the quantification of intracellular FMDV RNA synthesis relative to viral RNA release. Lipofection and electroporation were carried out as previously described [41], [62].

### Extraction of RNA, cDNA synthesis, PCR amplification, and nucleotide sequencing

RNA was extracted from the supernatants of infected cells by treatment with Trizol (Invitrogen) as previously described [63]. Reverse transcription (RT) was carried out using avian myeloblastosis virus reverse transcriptase (Promega) or Transcriptor reverse transcriptase (Roche), and PCR amplification was performed using *EHF* DNA polymerase (Roche) as specified by the manufacturer. PCR amplifications to obtain mutant infectious clones were carried out using *Pfu Turbo* DNA polymerase (Stratagene) because of its high copying fidelity [64]. Nucleotide sequencing was carried out as described [61].

### Plaque assays of viral samples

Plaque assays were performed as previously described [65]. Serial dilutions of viral samples were applied to confluent BHK–21 cell monolayers and incubated for 1 h at 37°C. Then, supernatants were removed and semisolid agar medium was added. Plaque development was permitted for 48 h post-infection.

### Plaque assay of directly transfected infectious RNA transcripts

Serial dilutions of *in vitro* transcribed infectious RNA (10, 1 and 0.1 ng) were mixed with lipofectin (Invitrogen) as indicated by the manufacturer. The mixture was added to a monolayer of 2×10^6^ BHK–21 cells and incubated for 2 h at 37°C. Then, the medium was removed, the cell monolayer was washed with DMEM and semisolid agar medium was added. Plaque development was permitted for either 48 or 72 h.

### Quantification of viral RNA in cell culture samples

Quantitative real time RT–PCR was carried out using the *Light Cycler RNA Master SYBR Green I* kit (Roche), according to the instructions of the manufacturer. Oligonucleotides spanning residues 3175 to 3194 (sense orientation) and 3518 to 3496 (antisense orientation), or 4924 to 4944 (sense) and 5026 to 5047 (antisense) were used in the amplification. Quantification was relative to a standard curve obtained with known amounts of FMDV RNA, synthesised by *in vitro* transcription of the infectious plasmid pMT28 [41]. For the quantification of extracellular FMDV RNA, the supernatants were collected, freed from detached cells by centrifugation, and extracted with Trizol (Invitrogen).

### Metabolic labelling of viral proteins

To detect viral protein synthesis in transfected cells, 2 to 8 µg of viral transcript were electroporated into 2×10^6^ BHK–21 cells. Electroporated cells were incubated for 3–4 hours in culture medium (DMEM, 1% FCS). Then, the medium was removed and cells were incubated for 1 h at 37°C in DMEM without Met and Cys, but supplemented with [^35^S] Met–Cys (Perkin–Elmer) (400 mCi/mmol). After metabolic labeling, cells were collected in 0.1 ml of sample buffer [160 mM Tris–HCl pH 6.8; 2% SDS; 11% glycerol; 0.1 M DTT, 0.033% bromophenol–blue]. The samples were heated at 90°C for 5 min and aliquots were subjected to SDS–PAGE (15% acrylamide).

### Western blot assays

Proteins were transferred to a 0.45 µm pore size nitrocellulose membrane (BioRad). Western blots were developed with the following antibodies at a dilution of 1∶2,000: mouse monoclonal anti–2C (1C8) and anti–3C (2D2) (a gift from E. Brocchi, Istituto Zooprofilattico Sperimentale della Lombardia e dell'Emilia Romagna, Brescia, Italy) and rabbit polyclonal anti–3D. Goat anti–rabbit IgG antibody coupled to peroxidase and goat anti–mouse IgG antibody coupled to peroxidase (Pierce) were used at 1∶10,000 dilutions. Each sample was analysed by Western blot to identify virus–specific proteins, following previously described procedures [62].
